# Applying a Total Market Lens: Increased IUD Service Delivery Through Complementary Public- and Private-Sector Interventions in 4 Countries

**DOI:** 10.9745/GHSP-D-15-00307

**Published:** 2016-08-11

**Authors:** Julia N White, Jamaica Corker

**Affiliations:** a Population Services International, Washington, DC, USA; b Independent Consultant, USA

## Abstract

Increasing access to the intrauterine device (IUD), as part of a comprehensive method mix, is a key strategy for reducing unintended pregnancy and maternal mortality in low-income countries. To expand access to IUDs within the framework of informed choice, Population Services International (PSI) has historically supported increased IUD service delivery through private providers. In applying a total market lens to better understand the family planning market and address major market gaps, PSI identified a lack of high-quality public provision of IUDs. In 2013, PSI started a pilot in 4 countries (Guatemala, Laos, Mali, and Uganda) to grow public-provider IUD service delivery through increased public-sector engagement while maintaining its ongoing focus on private providers. In collaboration with country governments, PSI affiliates carried out family planning market analyses in the 4 pilot countries to identify gaps in IUD service delivery and create sustainable strategies for scaling up IUD services in the public sector. Country-specific interventions to increase service delivery were implemented across all levels of the public health system, including targeted advocacy at the national level to promote government ownership and program sustainability. Mechanisms to ensure government ownership were built into the program design, including a proof-of-concept approach to convince governments of the feasibility and value of taking over and scaling up interventions. In the first 2 years of the pilot (2013–2014), 102,055 IUD services were provided to women at 417 targeted public-sector facilities. These preliminary results suggest that there is untapped demand for IUD service delivery in the public sector that can be met in part through greater participation of the public sector in family planning and IUD provision.

## Abstract (precis)

Between 2013 and 2014, IUD insertions for women increased more than threefold, from 22,893 to 79,162, in 417 public facilities in Guatemala, Laos, Mali, and Uganda through a Population Services International pilot that engaged the public sector alongside existing private-sector interventions. Based on family planning market analyses, the country-specific interventions focused on strengthening policy, service delivery, supply chain management, and demand creation.

## Teaser

Between 2013 and 2014, IUD services provided to women increased more than threefold—from 22,893 to 79,162—in 417 public facilities in Guatemala, Laos, Mali, and Uganda through a Population Services International pilot that engaged the public sector alongside existing private-sector interventions within an informed choice context. Based on family planning market analyses, the country-specific interventions focused on strengthening policy, service delivery, supply chain management, and demand promotion.

## BACKGROUND

Increasing access to long-acting reversible contraceptives (LARCs), including the intrauterine device (IUD), is a key global strategy for reducing unintended pregnancy and maternal mortality.^1^ IUDs are among the most effective forms of contraception,^2^^,^^3^ particularly compared with short-acting methods that are far more commonly used in most developing countries.^4^ IUDs are also among the most cost-effective and safest forms of contraception^5^ and can play an important role in helping governments to reach Millennium Development Goal 5 (reduce maternal mortality; universal access to reproductive health) and the Sustainable Development Goals.

The challenge, however, is that the IUD has historically been underrepresented in most family planning markets, with little support from the private or public sectors to supply the method and little willingness and capacity to offer services.^6^ On the supply side, the main challenge is that IUDs are provider-dependent and require that health care providers be trained and willing to perform insertions and removals. On the demand side, many women are hesitant to use the IUD because of the insertion procedure that they consider invasive and because of persistent safety and efficacy misconceptions.^7^^-^^9^ Other barriers to IUD uptake include low awareness of the method, greater availability of short-acting methods, and limited counseling from providers on LARCs.^10^^,^^11^

The IUD has been underrepresented in most family planning markets.

Population Services International (PSI) is a not-for-profit NGO operating in 66 countries around the world. Through network member organizations and network affiliates, PSI markets affordable health care products and services in developing countries and has maintained a focus on family planning by providing access to, promoting demand for, and improving service delivery of contraception.

As part of its family planning programs, PSI has prioritized expanding access to IUDs and establishing greater sustainability of IUD services in low-income countries within the context of informed choice.^5^ Informed choice means that women are able to make voluntary and informed decisions about family planning and have access to a full range of contraceptive methods. From 2008 through 2010, one of PSI’s approaches for expanding the method mix was to focus on IUDs where they were unavailable or difficult to access, while maintaining an ongoing focus on providing the full range of available contraceptive options. This approach relied heavily on vouchers, free services, mobile service delivery, and seconding providers to public clinics.

By the end of 2010, however, PSI began phasing out these activities in favor of approaches that rely less heavily on donor funding. PSI switched its focus to supporting networks of private providers with the training, quality assurance, and equipment to provide nonsubsidized IUD services.^5^ Targeted interventions included supportive supervision, on-the-job coaching and training, systematic quality audits, nonmonetary incentive schemes, and medical detailing. Improved interpersonal communication strategies, including careful mapping of intervention zones and use of education-through-listening techniques, helped boost consumer demand for IUDs and referrals to clinics for services. In addition, national-level advocacy aimed to foster a more supportive policy environment for IUD provision by focusing on establishing national LARC/IUD strategies, designing guidelines and curricula for health care provider trainings, and establishing specific country budget line items for LARC/IUD procurement. It was expected that these targeted interventions would increase high-quality private-sector service delivery while national-level advocacy would translate into increased government support of public-sector IUD service delivery, ultimately resulting in increased IUD provision nationally.

**FIGURE 1.**
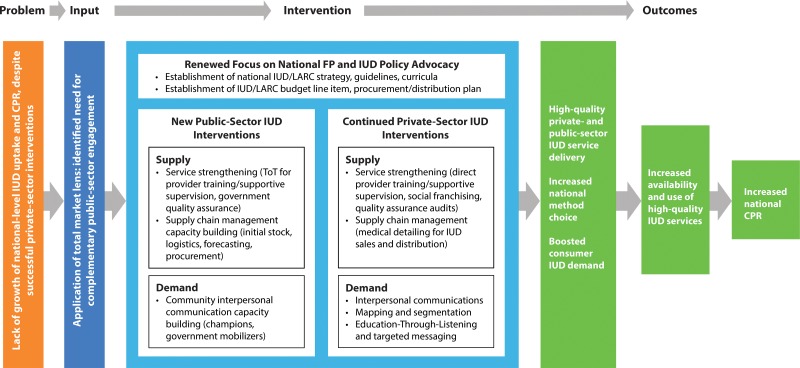
Population Services International’s Theory of Change for Implementing Complementary Public‐ and Private‐Sector IUD Interventions Abbreviations: CPR, contraceptive prevalence rate; FP, family planning; IUD, intrauterine device; LARC, long‐acting reversible contraceptive; ToT, training of trainers.

While there was initial growth in IUD uptake within the different country programs, PSI’s efforts rarely translated into measurable population-level increases in the contraceptive prevalence rate (CPR) or changes in method mix. To better understand the issue, PSI applied a total market lens to look across the multiple players involved in family planning and IUD supply and demand in PSI IUD-intervention countries and identify where the market was failing or had gaps. PSI found that even successful private-provider family planning networks were often too small to reach the large segment of women of reproductive age who seek health care in public facilities. In addition, advocacy undertaken for national-level policy and procurement changes had generally not translated into increased IUD service delivery at public facilities. PSI determined that a lack of high-quality *public* IUD service delivery was a barrier to growing IUD use and expanding method choice in order to increase the overall CPR. It is crucial to ensure high-quality service provision of IUDs across both the public and private sectors, as inconsistent quality of service delivery in any one sector of the market can drive down overall demand. In PSI’s theory of change (Figure 1), complementing its ongoing private-sector family planning and advocacy activities with a package of public-sector IUD capacity-building interventions would lead to increases in the uptake of IUDs that could ultimately impact the national-level CPR.

In PSI’s theory of change, complementing ongoing private-sector family planning and advocacy activities with public-sector IUD capacity-building interventions can lead to increases in IUD uptake.

In 2013, PSI began a public-sector engagement intervention to pilot the theory of change outlined above in 4 countries: Guatemala, Laos, Mali, and Uganda. PSI chose these countries for the pilot because they were geographically and contextually diverse and all had high unmet need and low IUD use (Table 1), despite a history of successful PSI private-sector IUD interventions.

**TABLE 1.** Unmet Need for Family Planning, Contraceptive Prevalence Rates, and Source of IUDs Among Married Women of Reproductive Age in PSI Pilot Countries, 2008–2013

	Guatemala 2008–2009	Laos 2011–2012	Mali 2012–2013	Uganda 2011
Unmet need	20.8%	19.9%	26.0%	34.3%
CPR for all modern methods	44.0%	42.1%	9.9%	26.0%
CPR for IUDs	1.3%	1.6%	0.4%	0.5%
CPR for implants	NA^a^	0.1%	2.5%	2.7%
IUD source				
Public sector	51.8%	80.2%	78.3%	38.9%
Private/NGO sector	46.2%	16.2%	21.7%	50.4%
Other sector/missing	2.1%	3.6%	0%	10.6%

Abbreviations: CPR, contraceptive prevalence rate; IUD, intrauterine device; PSI, Population Services International.

a Implants are included in the “other methods” category in the 2011 Guatemala survey.

Sources: Country household surveys.^12^^-^^15^

This article describes the first 2 years (January 2013–December 2014) of implementation in these 4 countries. It starts by detailing the country-specific analysis of family planning markets carried out to identify barriers to increasing IUD uptake. It then describes how each country designed interventions tailored to the public sector based on findings from the market assessment and calling on PSI's best practices from private-sector IUD programs. Last, it outlines ongoing scale-up and sustainability steps designed to transition responsibility of all activities to government partners.

## DATA SOURCES

Program data come from PSI country-level management information systems (MIS) from January 2011 through December 2014. Overall progress on this project is tracked through MIS data that are collected and reported monthly on IUD service delivery by distribution channel. Data for the market and facility analysis come primarily from baseline public facility assessments, PSI's own formative research studies, and regular in-country market analyses of family planning supply and demand. PSI collects and analyzes data according to international principles of maintaining privacy and confidentiality of personal information. These data are complemented with secondary sources, including the Demographic and Health Surveys^12^^-^^14^ and other household health survey data.^15^

## PROGRAM DESCRIPTION

The pilot implementation process was not rigidly defined from the start. Rather, PSI affiliates in the 4 pilot countries loosely followed similar steps, as defined below.

### Step 1: Understanding the Total Market

Prior to the start of the pilot, PSI affiliates in the 4 countries had been implementing successful private-sector IUD programs since 2011. PSI, therefore, already had an understanding of the family planning and IUD private-sector landscape in each country, including supply and demand barriers and gaps. To complement this knowledge, PSI encouraged its affiliates to apply a total market lens at the country level to look across the multiple players involved in family planning and IUD supply and demand and identify market failings or gaps, with particular attention to barriers in public-sector IUD service delivery. In collaboration with the country governments, PSI carried out family planning market analyses. PSI affiliates first gathered information on the CPR, the overall method mix, and modern method and LARC use. PSI also gathered more detailed information, including method source among current users, to identify where women in the respective countries were obtaining IUD services (Table 1).

Across all 4 countries, short-acting methods accounted for the majority of modern method CPR, while IUDs were used by only 0.4% to 1.6% of women of reproductive age (Table 1). Using these data, PSI affiliates worked with Ministry of Health (MOH) counterparts to forecast the impact of an expanded method mix, specifically one with an increased proportion of LARC and IUD use, on overall CPR growth. The MOH in all 4 countries expressed interest in working to expand the method mix through increased availability of IUDs.

As part of public-sector landscape analyses, PSI affiliates and the MOH conducted baseline assessments of preselected public-sector facilities to identify barriers to IUD uptake at the facility level. These baseline facility assessments included either a census of all eligible public facilities in intervention focus areas (Laos, Mali, and Uganda) or a survey of selected intervention public facilities (Guatemala). Data were gathered first from facility registers on levels of service delivery, and then interviews were conducted with facility staff to determine provider skill level and supply and equipment availability. These public facility assessments complemented PSI’s own formative research and regular in-country market analyses of family planning supply and demand that look at provider skill and motivation, commodity supply chain, consumer awareness, and availability of IUD insertion and removal equipment at facilities.

Findings from the market analyses pointed to both shared and unique gaps in the public provision of IUDs across the 4 countries (Table 2). Common supply and demand barriers were similar to those previously identified in the private sector in these and other countries: low consumer demand, misconceptions about IUD safety and effectiveness, low levels of provider skill and motivation, and inconsistent quality of service delivery. There were notable country-specific variations, however, that highlighted key areas to address in order to have the greatest potential impact on IUD uptake. For example, challenges in each country’s commodity supply chain varied from low levels of procurement at the national level in Guatemala to problems with commodity forecasting and monitoring that led to stock-outs at different subnational levels in Laos, Mali, and Uganda. The identification of these context-specific priorities in the public sector was crucial for guiding the later development of interventions with MOH partners.

There were notable country-specific variations that highlighted key areas to address in order to have the greatest potential impact on IUD uptake.

**TABLE 2.** Key Findings of the Pilot Country Baseline Market Assessments

	Service Delivery Quality	Commodity Supply Chain	Demand	Policy
Guatemala	IUD and infection prevention equipment available in maternity and district hospitals; limited PPIUD equipment and gaps in provider IUD/PPIUD skills	Low levels of IUD procurement at the national level	Some demand for interval IUD services; little demand for PPIUD services	LARCs not included in the national family planning strategy;PPIUD not in the national guidelines for hospital providers
Laos	Average of 8 years since last IUD training among public family planning providers	Challenges in provincial forecasting and delayed transport of contraceptives to the provincial level resulted in IUD stock-outs	72% of women had heard of IUDs; high prevalence of myths and misconceptions	2012 moratorium on IUD service delivery in the private sector;lack of national LARC strategy
Mali	59% of public-sector family planning providers in surveyed facilities had never inserted an IUD	Inconsistent IUD availability at the health center level	53% of women had never heard of IUDs; high reluctance to undergo an IUD procedure that they considered invasive	IUD service delivery restricted to doctors and selected midwives;lack of national LARC strategy
Uganda	65% of surveyed public facilities had at least 1 provider trained in IUD insertion; limited IUD insertion equipment	Lack of district supply monitoring mechanism linked to stock-outs at secondary health facilities	70% of women were aware of IUDs; low IUD demand attributed to myths and misconceptions	Inactive family planning commodity security working group

Abbreviations: IUD, intrauterine device; LARC, long-acting reversible contraceptive; PPIUD, postpartum IUD.

### Step 2: Designing Interventions to Address Supply and Demand Barriers

Beginning in 2013, PSI affiliates began working directly with government partners to develop public-sector IUD interventions that would respond to gaps identified in the market assessments and complement ongoing successful private-provider programs. Initial geographic intervention areas and facilities were strategically selected with government partners in each country to increase geographic coverage of IUD services by prioritizing areas with large populations, high unmet need, and public health facility infrastructure (Table 3).

**TABLE 3.** Geographic Intervention Areas With Supported Public Facilities, as of December 2014

Country	Geographic Intervention Areas	Supported Public Facilities (N = 417)^a^
Guatemala	17 of 22 departments	24
Laos	10 of 17 provinces^b^	82
Mali	4 of 9 regions	189
Uganda	20 of 111 districts	122

a The number of supported public facilities generally increased over time, with minor fluctuations between January 2013 and December 2014 due to intervention roll-out plans. Not all identified facilities began receiving support at the same time, and some facilities did not respond during data collection rounds conducted at multiple time points. The number of supported public facilities reported in this table and in the article is as of December 2014.

b Two fully covered intervention provinces, as well as limited intervention in 7 additional provinces (1 hospital per province) and several central hospitals in the capital.

From the market assessments and its previous private-sector IUD experience, PSI and its affiliates identified 4 broad intervention categories: policy, service strengthening, supply chain management, and demand promotion. Policy activities aimed to foster an enabling environment for increased public provision of IUDs and included continued advocacy to establish national strategies, guidelines, and curricula for LARCs/IUDs, and quantified government LARC/IUD procurement and distribution. Public-sector service strengthening activities included training of trainers to perform cascade trainings at all levels of the public sector and establishing systematic supportive supervision and MOH-led quality assurance audits for public facilities. Supply chain management involved providing initial commodity and equipment seed stock with technical assistance to strengthen commodity supply chain logistics, forecasting, and procurement. Demand-promotion activities most often included training public-sector providers in comprehensive family planning counseling, conducting interpersonal communication activities that included one-on-one or small-group conversations with women to better identify and respond to their family planning needs and concerns, and training public-sector communication agents to conduct the interpersonal communication activities. Within each country, PSI affiliates and the government collaborated to design specific interventions for each of the 4 categories, as outlined for individual countries below.

#### Guatemala: Pan American Social Marketing Association (PASMO/Guatemala)

In Guatemala, the baseline facility assessment found that postpartum IUD (PPIUD) services were generally not available in maternity wards, and external research confirmed that the IUD was the least offered and requested of all modern contraceptive methods in public hospitals after delivery.^16^ While most maternity hospitals and centers surveyed were well placed to offer PPIUD services, they lacked fully trained staff and complete equipment. In collaboration with district-level staff of the Ministry of Public Health and Social Welfare (MSPAS), PASMO/Guatemala designed a strategy to introduce PPIUD service delivery in 37 facilities in 30 targeted districts that had labor and delivery services but registered few postpartum IUD services.

- **Policy:** PASMO/Guatemala successfully advocated the inclusion of PPIUD insertion in the insertion protocols of the MSPAS National Guidelines for Family Planning, as well as the addition of PPIUD service delivery in the curriculum in 12 teaching hospitals.
- **Service** **strengthening:** In collaboration with district-level MSPAS staff, PASMO/Guatemala started implementation in 24 of the 37 eligible facilities. PASMO/Guatemala trained 10 MSPAS master trainers in IUD/PPIUD service delivery and provided follow-up supportive supervision to increase confidence. These master trainers are now responsible for organizing cascade trainings for MSPAS staff at secondary-level facilities in their districts.
- **Supply chain management:** In collaboration with the National Contraceptive Commodity Security Committee (CNAA), PASMO/Guatemala facilitated the development of a contraceptive commodity forecasting tool to advocate increasing the share of LARCs in the method mix and to help the government plan for contraceptive procurement.
- **Demand promotion:** PASMO/Guatemala trained public-sector and auxiliary nurses in demand promotion and family planning counseling and conducted community-level one-on-one and small-group interpersonal communication activities with women.

#### Laos (PSI/Laos)

Although Laos requires all district and larger hospitals to offer IUD services, the market assessment highlighted areas to strengthen the quality of public-sector service delivery. Borrowing best practices from a successful primary health care capacity-building model,^17^ PSI/Laos worked with the MOH to select 2 provinces (Vientiane Province and Champasak) in which to pilot the multilevel public-sector model for improving IUD service delivery. Interventions were designed to improve and increase IUD service delivery in the public sector by strengthening services in the district hospitals and introducing IUD services in selected health centers. To prepare for future scale-up of activities and strengthen provincial trainer skills, the strategy also included the provision of limited demand promotion and supervision support in 7 provincial hospitals and in the 4 central hospitals in the capital.

- **Policy:** Because of the 2012 government moratorium on private-sector IUD service delivery, PSI's policy work in Laos focused on advocating changes in the family planning law and the development of a national LARC strategy to allow for the resumption of IUD service provision by private providers.
- **Service strengthening:** PSI/Laos conducted an IUD training of provincial trainers, designed to establish cascade trainings down to the district hospital and health center levels that would include post-training supportive supervision.
- **Supply chain management:** PSI/Laos worked with the Medical Products Supply Technical Working Group to strengthen supply chain management for donated commodities. PSI/Laos and the United Nations Population Fund (UNFPA) agreed to sponsor a future workshop on estimating commodity needs with provincial maternal and child health coordinators in June 2015.
- **Demand promotion:** PSI/Laos interpersonal communication agents were assigned to each facility's catchment area to raise women’s awareness of family planning and knowledge of facilities offering family planning counseling. In addition, PSI/Laos created a new village-level network of family planning champions, who can also promote IUDs, to gradually assume responsibility for demand promotion work.

#### Mali (PSI/Mali)

The public-sector facility assessment in Mali identified a dearth of qualified IUD providers at community health centers, owing to restrictions on provider eligibility to perform IUD services. As a result, PSI/Mali and the MOH identified task shifting to allow IUD service delivery (previously limited to doctors and a limited number of midwives) by all midwives and obstetric nurses as a priority. To complement the task shifting, they also prioritized the need to work with regional departments and health districts to strengthen IUD services in the referral hospitals. In collaboration with the regional and district health offices, PSI/Mali selected target health centers in 3 intervention regions (Kayes, Ségou, and Sikasso) and the capital (Bamako), based on the population in the facility catchment area, geographic accessibility, availability of qualified staff, and infrastructure for infection prevention and patient privacy.

- **Policy:** At the national level, PSI/Mali successfully advocated approval to pilot the task shifting of IUD provision to midwives and obstetric nurses in lower-level public-sector facilities. PSI/Mali advocacy efforts also resulted in the inclusion of practical IUD training in the nursing and midwifery schools.
- **Service strengthening:** PSI/Mali supported the regional public-sector trainers to roll out IUD trainings and supportive supervision. Support was also given to public-sector district supervisors to conduct annual audits of the public-sector sites.
- **Supply chain management:** PSI/Mali helped organize quarterly district-level commodity forecasting meetings with health officials to improve methods for forecasting commodity needs.
- **Demand promotion:** In collaboration with local community-based organizations, PSI/Mali trained health center matrons (traditional birth attendants who provide health outreach) to incorporate LARCs/IUDs into their one-on-one or small-group outreach activities where they discuss reproductive health, including family planning.

#### Uganda: Programme for Accessible Health Communication and Education (PACE/Uganda)

In 2011, Uganda’s national CPR for any modern method among married women of reproductive age was 26.0%, and only 0.5% for IUDs.^13^ Prior to 2013, PACE/Uganda implemented family planning and IUD interventions through its private, urban franchise of family planning service providers (ProFam). To expand its reach as part of the public-sector engagement approach, PACE/Uganda decided to focus on public facilities in rural areas. PACE worked with the MOH to design a broad strategy for strengthening IUD service delivery in these public facilities through direct collaboration with government district health teams (DHTs) across the country. After meetings with the MOH, 20 districts from across the country were initially chosen based on high unmet need, the presence of secondary-level health centers, and the number of active district health officers. Notably, prior to beginning activities, PACE/Uganda signed a memorandum of understanding (MOU) with the MOH and with each intervention district. The district MOUs formalized roles and responsibilities for sustainability and scale-up, including goals for annual growth in the number of public facilities offering IUD services.

- **Policy:** At the national level, PACE/Uganda was instrumental in reviving the Family Planning/Reproductive Health (FP/RH) Commodity Security Working Group and collaborating with this working group to update the procurement tables for contraceptives in order to streamline government forecasting and procurement planning. In addition, PACE/Uganda worked with the government and key stakeholders on a National Costed Implementation Plan on Family Planning.
- **Service strengthening:** Per the MOUs, PACE/Uganda initially trained 1 provider per selected facility and 1 reproductive health focal person per district in IUD service delivery, with steps put in place to help the districts assume responsibility for implementation and costs over time. The DHT then designated master trainers to lead trainings and reproductive health focal persons to lead quarterly supportive supervision and quality assurance at supported public-sector facilities in their districts.
- **Supply chain management:** PACE/Uganda participated in quarterly FP/RH Commodity Security Technical Working Group meetings that monitored national family planning procurement, forecasted commodity needs, and coordinated partner efforts. PACE/Uganda also collaborated directly with DHTs to strengthen district IUD service planning, logistics, and budgeting.
- **Demand promotion:** PACE/Uganda facilitated DHT organization of monthly dialogue meetings with community health workers to help them address commonly encountered myths and misconceptions about family planning and IUDs. PACE/Uganda also supported family planning event days to promote family planning information and services, and to link women who wished to use the IUD immediately with providers with the most up-to-date training and skills.

**Figure:**
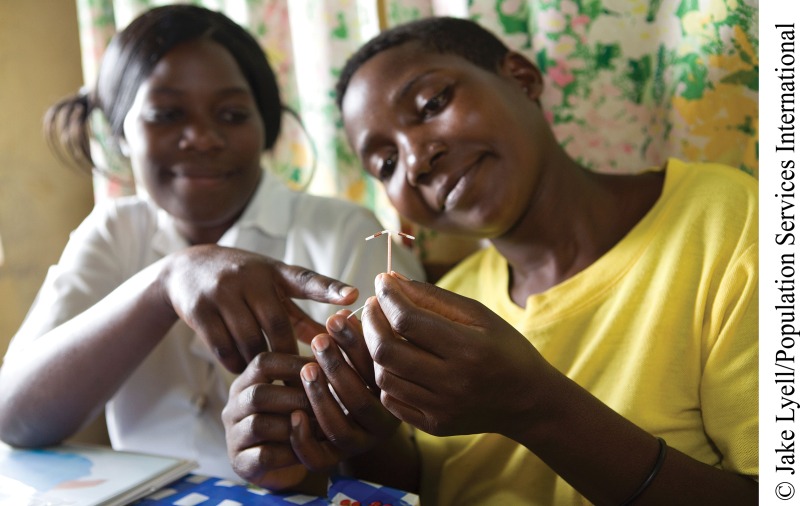
A family planning client at Kawoko Muslim Health Centre in Kawoko Town, Uganda, holds an IUD while a midwife counsels her about the method.

**FIGURE 2.**
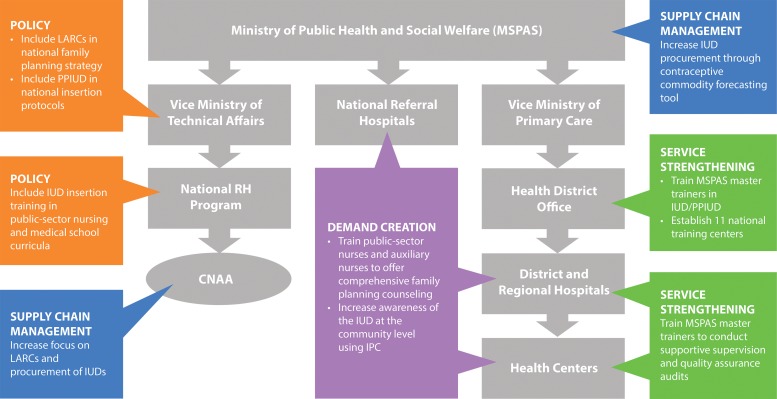
Intervention Strategy in the Public Health System in Guatemala to Increase Public Provision of IUDs Within an Informed Choice Context Abbreviations: CNAA, National Contraceptive Commodity Security Committee; IPC, interpersonal communication; IUD, intrauterine device; LARC, long‐acting reversible contraceptive; MSPAS, Ministry of Public Health and Social Welfare; PPIUD, postpartum intrauterine device; RH, reproductive health.

Across all 4 countries, integrating the intervention package into the public health care system required continual advocacy and ongoing capacity-building interventions at different levels of the health system in order to implement changes made in national policy. PSI had learned that policy change did not necessarily lead to improvements at the facility level without subnational intervention and, at the same time, facilities could often not make service delivery improvements without policy guidelines and curricula. As a result, the public-sector IUD interventions were designed to target all levels of the public health system concurrently. If service strengthening at the national level focused on revising health care provider training guidelines, for example, direct assistance was provided for cascade trainings, based on the new guidelines, down to district and community health facilities. Likewise, commodity forecasting and procurement at the national level was coupled with concurrent support to regional- and district-level government authorities to improve forecasting and ordering to avoid stock-outs. The concept was similar in all countries; as an example, Figure 2 illustrates the different levels of IUD-specific interventions in the Guatemala pilot.

Across all 4 pilot countries, PSI’s public-sector IUD interventions targeted all levels of the public health system concurrently.

### Step 3: Planning for Sustainability and Scale-Up

In all 4 pilot countries, PSI worked directly with government officials at the national and subnational levels to negotiate mechanisms in the intervention design to ensure eventual government ownership of increased IUD service delivery. PSI and government partners in each country agreed on a proof-of-concept approach, whereby PSI would provide initial intensive investment to support public-sector IUD interventions in a small geographic area, and would then use preliminary results to determine with the government the feasibility and value of scaling up the interventions nationally. Inherent in this approach was a gradual and structured transfer of implementation and financial support responsibilities. Each year, government ownership of the national IUD program was expected to grow as PSI support to previous areas was reduced and instead focused on new areas.

**FIGURE 3.**
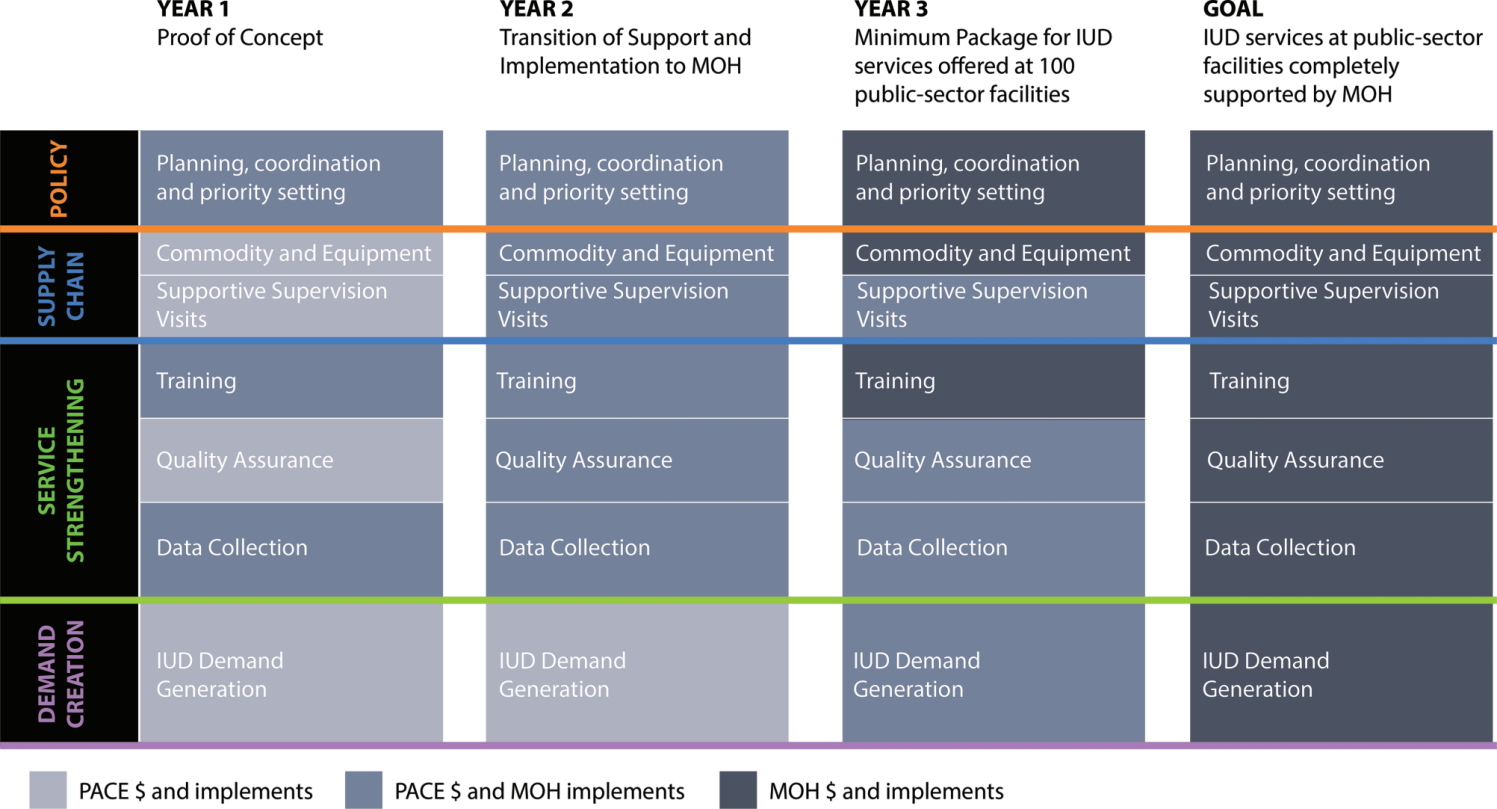
Uganda Sustainability and Scale‐Up Plan to Transition the Public-Sector IUD Intervention Package to the Ministry of Health Abbreviations: IUD, intrauterine device; MOH, Ministry of Health; PACE, Programme for Accessible health Communication and Education.

The 4 PSI affiliates are working to design scale-up and sustainability plans that outline key activities and specify when primary responsibility for program costs and implementation will shift from PSI affiliates to MOH partners. Development of these plans is ongoing and specific to each country, although PSI’s experience in Uganda offers a more advanced example: PACE/Uganda's early focus on national- and district-level MOUs facilitated a fluid transition from distinct roles and responsibilities to creating a sustainability and scale-up plan for some of the initial interventions (Figure 3). PSI affiliates in the other pilot countries are internally defining similar sustainability and scale-up plans.

## PRELIMINARY RESULTS

After initial implementation of the IUD public-sector engagement interventions (January 2013 to December 2014), there were notable increases in IUD uptake in pilot intervention areas across all pilot countries (Table 4): 22,893 IUD services were provided to women in the first year of implementation, increasing more than threefold to 79,162 IUD services in the second year. Thus, the total number of IUD services provided was 102,055 in the first 2 years. By the end of 2014, PSI affiliates were working through the respective country ministries of health to implement new packages of support activities for IUD service delivery in 417 public-sector facilities that previously offered limited IUD services. In the private sector, between 2011 (when IUD private-sector interventions began in the 4 pilot countries) and 2014, private-sector networks in the 4 pilot countries increased annual IUD services provided to women by almost 100%, from 32,127 to 59,716 (Table 4).

**TABLE 4.** IUD Insertions by PSI-Supported Private- and Public-Sector Providers by Country, 2011–2014

Country	Private-Sector Providers					Public-Sector Providers				
	2011	2012	2013	2014	Country Total	2011^a^	2012^a^	2013	2014	Country Total
Guatemala	6,876	11,876	12,607	14,831	**46,190**	-	-	830	3,719	**4,549**
Laos^b^	240	657	199	0	**1,096**	-	-	906	6,812	**7,718**
Mali	1,608	5,999	8,326	8,219	**24,152**	-	-	8,662	9,906	**18,568**
Uganda	23,403	45,589	40,573	36,666	**146,231**	-	-	12,495	58,725	**71,220**
**Annual total**	**32,127**	**64,121**	**61,705**	**59,716**	**217,669**	-	-	**22,893**	**79,162**	**102,055**

a Figures for 2011 and 2012 were not available.

b A 2012 government moratorium on private-sector IUD service delivery halted private-provider activities.

## DISCUSSION AND PROGRAM IMPLICATIONS

Preliminary results from the first 2 years of PSI's public-sector engagement pilot seem to offer support for the theory of change that complementing private-sector family planning activities with public-sector IUD capacity-building interventions can lead to increased provision of IUDs. The rapid increase in IUD service delivery among newly added public facilities, coupled with consistently strong IUD service delivery among PSI-supported private-sector providers, indicate that there is untapped demand for IUDs in both sectors in these 4 pilot countries. PSI believes that robust results from the first 2 years of program implementation, much of which involved start-up activities, suggest that continued growth in both sectors could lead to increases in IUD uptake that may help to increase the national-level CPR.

PSI also believes that encouraging early growth in public-sector IUD service delivery in these 4 pilot countries was due in part to the initial and sustained collaboration with governments on the design and implementation of intervention activities. Government collaboration took different forms across the pilot countries, but in all cases involved joint selection of priority geographic areas and co-design of country-specific public-sector intervention activities to address priority areas of IUD unmet demand. Engaging MOH partners from the earliest stages of the project also appears to play an instrumental role in establishing sustainability and scale-up plans to incrementally transfer the majority of roles and responsibilities to government partners.

Encouraging growth in public-sector IUD service delivery was due in part to the initial and sustained collaboration with governments on the design and implementation of intervention activities.

Additionally, PSI's previous experience in these countries facilitated the design of context-specific interventions. As a result of its presence in all 4 countries, PSI already had experience with each country's family planning landscape and government partners prior to the pilot interventions. With findings from the market assessments, PSI and its government counterparts were able to identify key barriers quickly and found that they aligned with the 4 intervention categories that PSI was already using in the private sector (policy, service strengthening, supply chain management, and demand promotion). Although many of the activities under this pilot involved creating new approaches specific to the public sector, certain best practices were also integrated from PSI's array of private-sector family planning and IUD program experience. PSI found, for example, that many demand-side barriers were similar in the public and private sectors and was able to apply some of its existing interpersonal demand-promotion interventions in the public sector. Additionally, given limited private- and public-provider motivation to offer IUD services, PSI worked to incorporate successful private-sector supportive supervision approaches into the public health system, with the aim of coaching providers to understand the value of offering a complete family planning package, including IUDs.

Last, findings from the early stages of this pilot also highlight the importance of working simultaneously across different levels of the government health system when promoting increased public-sector engagement in new intervention areas. National policy changes alone do not guarantee that these changes will be carried out at the facility level, and public health facilities often cannot implement improvements or changes without direct policy guidelines and curricula. As a result, PSI found that it was important to specifically design its public-sector IUD interventions to concurrently target all levels of the public health system, reinforcing national-level policy changes with capacity building at the subregional and district levels that responded directly to countrywide policy changes. This approach also allows feedback loops for top-down and bottom-up communication, as regional and district personnel are often involved in trainings and activities at the local level and with working groups at the national level, which enables them to promote improved IUD service delivery nationally and locally.

## CONCLUSION

Complementing private-sector IUD activities with increased public-sector IUD interventions offers an opportunity to increase inclusion of IUDs as part of a comprehensive method mix. This is true particularly in countries that have strong private-sector service delivery interventions. The rapid growth in IUD service delivery in PSI's targeted public facilities in the 4 pilot countries in the first 2 years of program implementation, in tandem with consistently high numbers of IUD services provided to women by private providers, suggests that untapped demand for family planning can be met in part through greater participation of the public sector.

## Notes

**Peer Reviewed**
